# Evaluation of pre-processing on the meta-analysis of DNA methylation data from the Illumina HumanMethylation450 BeadChip platform

**DOI:** 10.1371/journal.pone.0229763

**Published:** 2020-03-10

**Authors:** Claudia Sala, Pietro Di Lena, Danielle Fernandes Durso, Andrea Prodi, Gastone Castellani, Christine Nardini

**Affiliations:** 1 Department of Physics and Astronomy, University of Bologna, Bologna, Italy; 2 Department of Computer Science and Engineering, University of Bologna, Bologna, Italy; 3 Division of Infectious Diseases and Immunology, University of Massachusetts Medical School, Worcester, Massachusetts, United States of America; 4 Smart Cities Living Lab, Institute of Organic Synthesis and Photoreactivity, CNR, Bologna, Italy; 5 Department of Experimental, Diagnostic and Specialty Medicine, University of Bologna, Bologna, Italy; 6 Interdepartmental Center “L. Galvani”, University of Bologna, Bologna, Italy; 7 Department of Laboratory Medicine, Karolinska Institutet, Stockholm, Sweden; 8 CNR IAC “Mauro Picone”, Roma, Italy; 9 Sol Group, Monza, Italy; Chuo University, JAPAN

## Abstract

**Introduction:**

Meta-analysis is a powerful means for leveraging the hundreds of experiments being run worldwide into more statistically powerful analyses. This is also true for the analysis of omic data, including genome-wide DNA methylation. In particular, thousands of DNA methylation profiles generated using the Illumina 450k are stored in the publicly accessible Gene Expression Omnibus (GEO) repository. Often, however, the intensity values produced by the BeadChip (raw data) are not deposited, therefore only pre-processed values -obtained after computational manipulation- are available. Pre-processing is possibly different among studies and may then affect meta-analysis by introducing non-biological sources of variability.

**Material and methods:**

To systematically investigate the effect of pre-processing on meta-analysis, we analysed four different collections of DNA methylation samples (datasets), each composed of two subsets, for which raw data from controls (i.e. healthy subjects) and cases (i.e. patients) are available. We pre-processed the data from each dataset with nine among the most common pipelines found in literature. Moreover, we evaluated the performance of *regRCPqn*, a modification of the RCP algorithm that aims to improve data consistency. For each combination of pre-processing (9 × 9), we first evaluated the between-sample variability among control subjects and, then, we identified genomic positions that are differentially methylated between cases and controls (differential analysis).

**Results and conclusion:**

The pre-processing of DNA methylation data affects both the between-sample variability and the loci identified as differentially methylated, and the effects of pre-processing are strongly dataset-dependent. By contrast, application of our renormalization algorithm *regRCPqn*: (i) reduces variability and (ii) increases agreement between meta-analysed datasets, both critical components of data harmonization.

## Introduction

The public availability of thousands of human DNA methylation datasets offers the possibility to gain appropriate statistical power to test hypotheses relevant to the mechanisms involved with methylation, to reveal and validate its role in health and disease and to identify stable epigenetic signatures, via meta-analysis. However, together with the opening of great opportunities, meta-analysis also brings critical challenges associated to data harmonization. This is particularly true for DNA methylation, where data are often available only in pre-processed rather than raw forms and where numerous pre-processing pipelines exist.

The popular international public repository Gene Expression Omnibus (GEO, [1]) contains over 100000 human DNA methylation samples, mostly measured using the Illumina Infinium HumanMethylation450 BeadChip [2] technology (GEO accession number GPL13534) (see Table 1).

**Table 1:** Summary of human DNA methylation data available on GEO listed by sequencing technology on 01/03/2019.

Technology (GEO accession number)	Year	Num of Series	Num of Samples
Illumina Infinium HumanMethylation27 BeadChip (GPL8490)	2009	340	18783
Illumina Infinium HumanMethylation450 BeadChip (GPL13534)	2011	1205	84898
Illumina Infinium MethylationEPIC BeadChip (GPL21145)	2015	100	3397
Bisulphite high throughput sequencing	−	31	18

The Illumina 450K BeadChip contains 485577 probes targeting 99% of RefSeq genes, besides several other locations on the genome [2]. Overall, the 450k probes target both CpG sites that are isolated on the genome (i.e. CpG OpenSeas) and CpG sites that reside in regions of variable density (CpG Islands, Shelves or Shores), targeting 96% of the CpG Island regions. Unlike the previous Illumina Infinium HumanMethylation27 BeadChip, this array incorporates two different chemical assays: Infinium I, that includes 135501 probes, and Infinium II, that includes 350076 probes. Each CpG site of Infinium I is targeted by two probes that respectively detect *methylated* (*M*) and *unmethylated* (*U*) signal intensities, while each CpG site of Infinium II is targeted by a single probe with 2 different dye colors (green and red) that distinguish *M* and *U* signal intensities.

The pre-processing pipeline designed to obtain methylation profiles from raw experimental data (.idat files) in the form of *β*-values (*β* = *M*/(*M* + *U* + *α*), where typically *α* = 100 [3]) is not standardized, even within the same technological platform, as illustrated in Table 2. Indeed, different pre-processing methods make different assumptions on the data regarding their distribution, their correlation structure and the extent (localized or global) of the DNA methylation variability on the genome [4], since this is still a matter of debate [5].

**Table 2:** Summary of the pre-processing methods considered in the study.

	SWAN	BMIQ	noob	FunNorm	dasen	pQuantile	RCP
**Background adjustment**	No	Yes	Yes	No	Yes	No	Yes
**Variables**	type I/II	type I/II	dye bias	sex M/F, control probes	type I/II	type I/II, sex M/F	type I/II, local correlation
**Genomic regions**	No	No	No	No	No	Yes	Yes
**Statistical method**	Subset quantile norm.	3 state *β*−mixture quantile norm.	Normal-exponential quantile norm.	Functional norm.	*β*−mixture quantile norm.	Stratified quantile norm.	Quantile norm.
**Normalization type**	within array	within array	within array	between array	between array	between array	within array

Pre-processing methods considered in the study, detailed by: background adjustment; variables considered such as probe type, subset of probes, sex or fluorescence color channel; genomic region; underlying statistical approach; normalization type. In addition, *NOOB* + *BMIQ* and raw *β*-values pipelines were obtained applying two of the above in sequence and considering unprocessed values, respectively, for a total of nine pre-processing methods.

Notice that the pre-processing transformations of the data aim at reducing the experimental variability but may also affect the biological variation of the samples. The magnitude of such undesired effect depends on the pre-processing method that was applied and remains a critical and ambiguous point in DNA methylation data analysis that will not be addressed here.

Besides the peculiarities of each pre-processing method, it is possible to identify a number of common computational manipulations applied to the raw data. These include probe type and colour bias adjustment, background signal subtraction and average normalization to reduce the effects of experimental variation early in the pipeline; and intra- and inter-sample normalization as well as batch effect correction, that are usually taken into consideration further down in the pipeline [6, 7].

When analysing a single dataset, the choice of the pre-processing pipeline must be done carefully to maximize the statistical power of the analysis and the robustness of the results [8]. In meta-analysis two further critical issues arise: dataset and sample selection, and also data harmonization. Meta-analysis can be performed in different ways: some approaches first analyse separately individual datasets and then combine the results into a final estimate; others first pool data from all the datasets and then analyse the pooled data using a single model. The first class of approaches includes *Aggregated Data* (*AD*) and *two-step Individual Participant Data* (*IPD*) meta-analyses [9]. The main advantage of these approaches is the relatively low complexity of their implementation. The main drawback is the need for raw data. The second class of approaches is referred to as *one-step IPD* meta-analysis. Although *one-step IPD* approaches are expected to behave similarly to the *two-steps IPD* ones [9], they allow additional flexibility (e.g. no need to start from raw data) and, for example, give the possibility to compare different models and investigate interactions [9]. An important assumption of the *one-step IPD* meta-analyses is the comparability of the variables measured in different datasets [10], and this is why data harmonization is crucial to guarantee that methylation samples of the same type (same tissue, health state, age, sex, etc.) from different datasets can be compared.

Knowing that less than half of the Illumina BeadArrays datasets in GEO include raw data (521 over 1205 studies considering the Illumina 450K BeadChip) we here propose a *one-step IPD* approach to systematically assess the effects of different pre-processing methods on meta-analysis.

Finally, we introduce *regRCPqn* (regional Regression on Correlated Probes with quantile normalization), as a possible solution (and currently the only one, to the best of our knowledge) to reduce *a posteriori* the variability and compensate for the artefacts in the *β*-value distribution resulting from different choices of pre-processing protocols. Enhancing existing methods (*RCP* [11]), *regRCPqn* enables meta-analyses even when raw data are not publicly available. To validate our method, we propose differential analysis as a ubiquitous and flexible approach to show the results of our findings.

## Materials and methods

To evaluate the effects of pre-processing on the between-sample variability and on the identification of Differentially Methylated Positions (DMPs) in case-control studies, we analyse four different collections of datasets. The datasets have been selected based on their sample size and on the assumption that there is no global DNA methylation variation among samples (see Introduction and [5]). This assumption is required to apply methods that are based on quantile normalization [4, 6]. In the following, we first describe the datasets (Section *Materials*) and the nine pre-processing methods that we tested (Section *Pre-processing*). We then introduce *regRCPqn* (Section *Regional RCP with quantile normalization* (*regRCPqn*)), and finally, we specify how the datasets are pre-processed (Section *Datasets pre-processing*), as well as which model and variables are used for the differential analysis (Section *Statistical Analysis*).

### Materials

We consider four datasets, each composed of two studies, characterized by features that are progressively challenging from the harmonization point of view: the first one (BloodIBD) is a dataset from a single project and laboratory, where samples are processed at two different dates, mimicking two datasets; the second one (BloodParkinson) artificially breaks one study into two batches; the third (NasalAsthma) and the fourth (BuccalFetalAlcohol) datasets represent real life scenarios, where independent projects address the same clinical issue (atopic asthma and foetal alcohol spectrum disorder (FASD), respectively) in different laboratories, different dates, different clinical centres and heterogenous patients enrolment. In all cases, the datasets raw.idat files were available in GEO.

1. BloodIBD: the dataset from [12], GSE87648, includes whole blood DNA methylation data from 384 samples (18-79 y.o.), 204 subjects with Inflammatory Bowel Disease (IBD) and 180 healthy controls. Samples were processed in two different dates: 192 samples (90 IBD and 102 Controls) were scanned on 11/13 (*date1*), while 192 samples (90 IBD and 102 Controls) were scanned on 11/20 (*date2*). Here we simulate a meta-analysis by considering the two batches (dates) as two distinct studies with limited experimental variation, since subjects were selected according to the same criteria and samples were sequenced in the same laboratory and with the same protocols.
2. BloodParkinson: the dataset from [13], GSE111629, includes whole blood DNA methylation data from 563 samples (35-92 y.o.), 329 subjects with Parkinson’s disease (PD) and 234 from healthy PD-free control subjects. Here the presence of two studies was simulated by artificially dividing the samples into two groups through a stratified sampling based on health status. One study includes 281 subjects (164 PD and 117 controls), while the other includes 282 subjects (165 PD and 117 controls). No batch effect is expected to exist between the two simulated studies, but we still use different pre-processing methods to investigate their effects on the results.
3. NasalAsthma: in this meta-analysis we consider two datasets that include DNA methylation data of nasal epithelial cells from children with persistent atopic asthma and controls. The first dataset (GSE65163) includes 72 subjects (9-12 y.o.), 36 asthmatics and 36 controls. The second dataset (GSE109446) includes 58 subjects (5-18 y.o.), 29 asthmatics and 29 controls.
4. BuccalFetalAlcohol: in this meta-analysis we consider two datasets that include DNA methylation data of buccal epithelial cells from children with FASD and controls. The first dataset (GSE80261 [14]) includes 202 subjects (5-18 y.o.), 106 FASD and 96 controls, that were processed in two different dates, 89 on 04/12 (44 FADS and 45 controls) and 113 on 06/13 (62 FADS and 51 controls). The second dataset (GSE109042 [15]) includes 54 subjects (4-18 y.o.), 26 FASD and 27 controls.

Raw data of each study (.idat files) were downloaded from the GEO database and pre-processed with the R packages minfi 1.28.0 [6], wateRmelon 1.26.0 [7] and ENmix 1.18.1 [16]. In all cases, samples and probes were first filtered according to the recommended criteria [6] and then pre-processed. Specifically, samples were discarded if > 5% of the probes had detection p-value > 0.05. Probes were filtered out if the detection p-value was > 0.05 in > 1% of the samples. Samples whose median intensities in both the methylated (*M*) and unmethylated (*U*) channels were lower than most cases were also removed. For the BloodIBD dataset, 256 samples over 384 were finally retained: 111 (60 IBD and 51 Controls) scanned on *date1* and 154 (87 IBD and 67 Controls) scanned on *date2*. For the BloodParkinson dataset all samples passed the pre-processing filters in both simulated batches. For the NasalAsthma meta-analysis 3 samples were discarded from GSE65163 and none from GSE109446. For the BuccalFetalAlcohol study 18 samples were removed from GSE80261, leaving 94 FASD and 90 controls, while 22 samples were removed from GSE109042, leaving 16 FASD and 16 controls.

### Methods

Raw data were pre-processed using eight of the most commonly used algorithms in literature [8]: 1) subset-quantile within array normalization (*SWAN*) [17], 2) Functional normalization (*FunNorm*) [4], 3) *dasen* [7], 4) stratified quantile normalization (*pQuantile*) [6], 5) normal-exponential using out-of-band probes (*noob*) [18], 6) Regression on Correlated Probes (*RCP*) [11], 7) Beta-Mixture Quantile Method (*BMIQ*) [19], and 8) the combination of *noob* and *BMIQ*, that was proven to outperform the other methods in [8].

Although a careful review of all methods can be found in [8], we here briefly recall their main features, further summarized in Table 2. In short, SWAN [17] performs within-array normalization separately for the methylated and unmethylated channels. First, it selects a random subset of type I and type II probes matched on the number of underlying CpG and it performs a quantile normalization. Then, it uses a linear interpolation to adjust the intensities of the remaining probes, separately for each probe type.

BMIQ [19] is a within-array normalization method that adjusts for probe type bias. It separately fits the *β*-values distribution of type I and type II probes with a three-state beta mixture model, where the three states correspond to unmethylated, 50% methylated and fully methylated probes. Then, *BMIQ* quantile normalizes type II probes by state on the distribution of type I probes of the same state.

*Noob* [18] is a within-array normalization method that first performs background subtraction by estimating the background mean intensity from the out-of-band control probes using a normal-exponential convolution model, and then normalizes the background-corrected intensities of the red and green channels based on the positive control probes.

Differently from the previous methods, *dasen* [7] performs a between-sample normalization. It equalizes type I and type II backgrounds and then quantile normalizes methylated and unmethylated intensities individually, considering separately type I and type II probes.

*FunNorm* [4] is also a between-sample normalization that uses the internal control probes to estimate and adjust for technical variations. It normalizes separately the two probe types, and the methylated/unmethylated signals. Moreover, it processes separately for males and females the probes that are located in chromosome X or Y. *FunNorm* is particularly suitable when global changes of the methylome are expected between samples (i.e. in cancer-control studies or when comparing the DNA methylation of different tissues), as it does not rely on the assumptions of quantile normalization.

Stratified quantile normalization (*preprocessQuantile*, here *pQuantile*) [6] is a between-sample normalization that takes into account the probe genomic region, known to affect the DNA methylation. It first quantile normalizes type I and type II intensities and then interpolates a reference distribution against which type I probes are normalized. This is done separately in each region and also separately for males and females for probes located in chromosome X and Y. Background correction is not used, but very small intensities are identified as outliers and removed.

Finally, *RCP* [11] is a within-array normalization that exploits the spatial correlation of DNA methylation on CpG sites. Namely, it uses type I and type II probes pairs that are closer than 25 base pairs and that share the same genomic context (Island, N_Shelf, N_Shore, S_Shelf, S_shore or OpenSea) to estimate the calibration transformation between type I and type II intensities.

In addition to the eight normalizations we also assessed raw data. In all cases, M-values (logit2 of *β*-values) rather than *β*-values were used for further analysis, as suggested in [20].

### Regional RCP with quantile normalization (*regRCPqn*)

Given the impact of heterogeneous normalizations on meta-analysis (see section Results and discussion), we propose an additional step to harmonize datasets that were pre-processed with different algorithms. Notice that our method does not require the raw.idat files and can hence be applied also to datasets with only pre-processed *β*-values available, a common real-life scenario. Our procedure, *regRCPqn*, outlined in Algorithm 1, specifically enhances *RCP* with the inclusion of three features to address the issue under study (i.e. meta-analysis from possibly differently pre-processed datasets). First (lines 4-8 in Algorithm 1), it computes the *RCP* normalization separately for each genomic region type (i.e. for CpG belonging to Islands, Shores, Shelves or OpenSeas), as the distribution of DNA methylation values is different in each of these regions [21] (RCP considers the regions to compute the transformation, but it does not normalize separately the CpG that belong to different regions). Then, it performs a between-sample quantile normalization where the CpG values of all samples are quantile normalized separately for each CpG region and for type I and type II probes (lines 11-12). Finally, it introduces the possibility to save a reference distribution and to use it to perform a reference-based quantile normalization of the samples from the other dataset(s) (lines 14-17). The reference distribution is computed separately for each region type and for type I and type II probes and it is computed as the average of the values obtained over the samples. When available, such distribution is used by *regRCPqn* to perform the reference-based normalization, again separately for each region and probe type (lines 21-22).

Notice that, as it is the case for some pre-processing methods, e.g. *dasen* and *pQuantile*, the *regRCPqn* algorithm performs a between-sample quantile normalization. Hence it should not be applied when global differences are expected, such as in cancer-control studies or when comparing the DNA methylation of different tissues. The X- and Y-chromosomes are removed from the analysis to avoid gender bias [22].

*regRCPqn* is implemented in R 3.5.1. It is available as open source R package on GitHub (https://github.com/regRCPqn/regRCPqn) and can be installed using the devtools command install_github(“regRCPqn/regRCPqn”).

**Algorithm 1** *regRCPqn*

**Require**: Mdata is a matrix of M-values; annot450k is a matrix of CpG region annotations that includes CpG region and probe type; refPDF = {refPDF.I, refPDF.II} are (optional) reference distributions of type I and II

1: **function** regRCPqn(Mdata, annot450k, refPDF)

2:  Mdata_out ← Mdata

3:  **for** R ∈ {OpenSea,Island,Shelve,Shore} **do**

4:   CpG_*R*_ ← CpG in region R from annot450k

5:   probe.I ← type I CpG_*R*_

6:   probe.II ← type II CpG_*R*_

7:   Mdata_*R*_ ← Mdata[GpG_*R*_]         ▹ Submatrix in region R

8:   RCP(Mdata_*R*_)            ▹ Run RCP on region R data

9:   **if** refPDF = NULL **then**

10:              ▹ Quantile normalization for each probe type

11:    Mdata_*R*_[probe.I] ← QN(Mdata_*R*_[probe.I])

12:    Mdata_*R*_[probe.II] ← QN(Mdata_*R*_[probe.II])

13:              ▹ Save reference distribution on file

14:    refPDF.I ← mean(Mdata_*R*_[probe.I])

15:    refPDF.II ← mean(Mdata_*R*_[probe.II])

16:    refPDF ← {refPDF.I, refPDF.II}

17:    save(refPDF)

18:   **else**

19:               ▹Quantile normalization for each probe type

20:               ▹wrt reference refPDF = {refPDF.I, refPDF.II}

21:    Mdata_*R*_[probe.I]←QN(Mdata_*R*_[probe.I],refPDF.I)

22:    Mdata_*R*_[probe.II]←QN(Mdata_*R*_[probe.II],refPDF.II)

23:   **end if**

24:               ▹Save region R normalized data

25:   Mdata_out[GpG_*R*_,] ← Mdata_*R*_

26:  **end for**

27:  **return** Mdata_out

28: **end function**

### Datasets pre-processing

We pre-processed the two studies of each dataset considering each possible combination of the nine pre-processing methods (eight pre-processing pipelines plus raw data). Then, we evaluated the effect of pre-processing on meta-analysis by performing a differential methylation analysis using the health status as variable of interest. In each meta-analysis we always considered the case of data re-normalized with *regRCPqn*. When *regRCPqn* was used, we first applied the re-normalization to one of the two studies (data from 11/13 for BloodIBD, first random subset for BloodParkinson, GSE65163 for NasalAsthma and GSE109042 for BuccalFetalAlcohol), and then we used the obtained reference distribution to normalize the samples from the other study. Before proceeding with the differential analysis we performed batch effect correction using Combat [23].

### Statistical analysis

We perform differential analysis to identified differentially methylated positions (DMPs) between cases and controls by computing a linear regression model for each CpG site. The analysis was performed in R 3.5.1. Each DNA methylation value (*M*-value) was regressed against the health status, while adjusting for confounding variables, an approach widely used in literature [12–15]. In BloodIBD, the confounding variables included age, sex, smoking status and cell counts, estimated with minfi [24]. In BloodParkinson and BuccalFetalAlcohol, smoking status was not available, but we included ethnicity. In BuccalFetalAlcohol we constrained the analysis to the 648 CpG sites that were investigated by the authors [15]. The NasalAsthma dataset GSE109446 included pairs of siblings, hence we introduced a random effect to model the correlations among family members. We fitted the model using the R libraries lme4 1.1.19 [25] and lmerTest 3.1.0 [26] and we included age, sex and ethnicity as confounding variables.

In all cases, statistical significance was set at p-value<0.05 following adjustment for multiple testing using the Benjamini-Hochberg correction.

Finally, we computed the variability in the number of DMPs obtained by the different pre-processing combinations as the Median Absolute Deviation (MAD). To test whether the MADs obtained using *regRCPqn* are lower than those obtained without *regRCPqn* harmonization, we performed a bootstrap resampling with 1000 iterations. Then, we tested for statistical significance using a one-tailed t-test. Finally, we identified outliers as points whose absolute distance from the median is larger than three times the dataset MAD.

## Results and discussion

### Between-sample variability

To investigate the impact of pre-processing on the distribution of M-values we estimated the global between-sample variability. For each meta-analysis, we considered all the control subjects (from both subsets), and we computed the standard deviation (SD) of each CpG site (M-value). Finally, we used the average SD as a measure of between-sample variability. The computation was performed for each of the nine pre-processing procedures outlined above, considering all combinations, including same pre-processing method for both studies.

**Fig 1:**
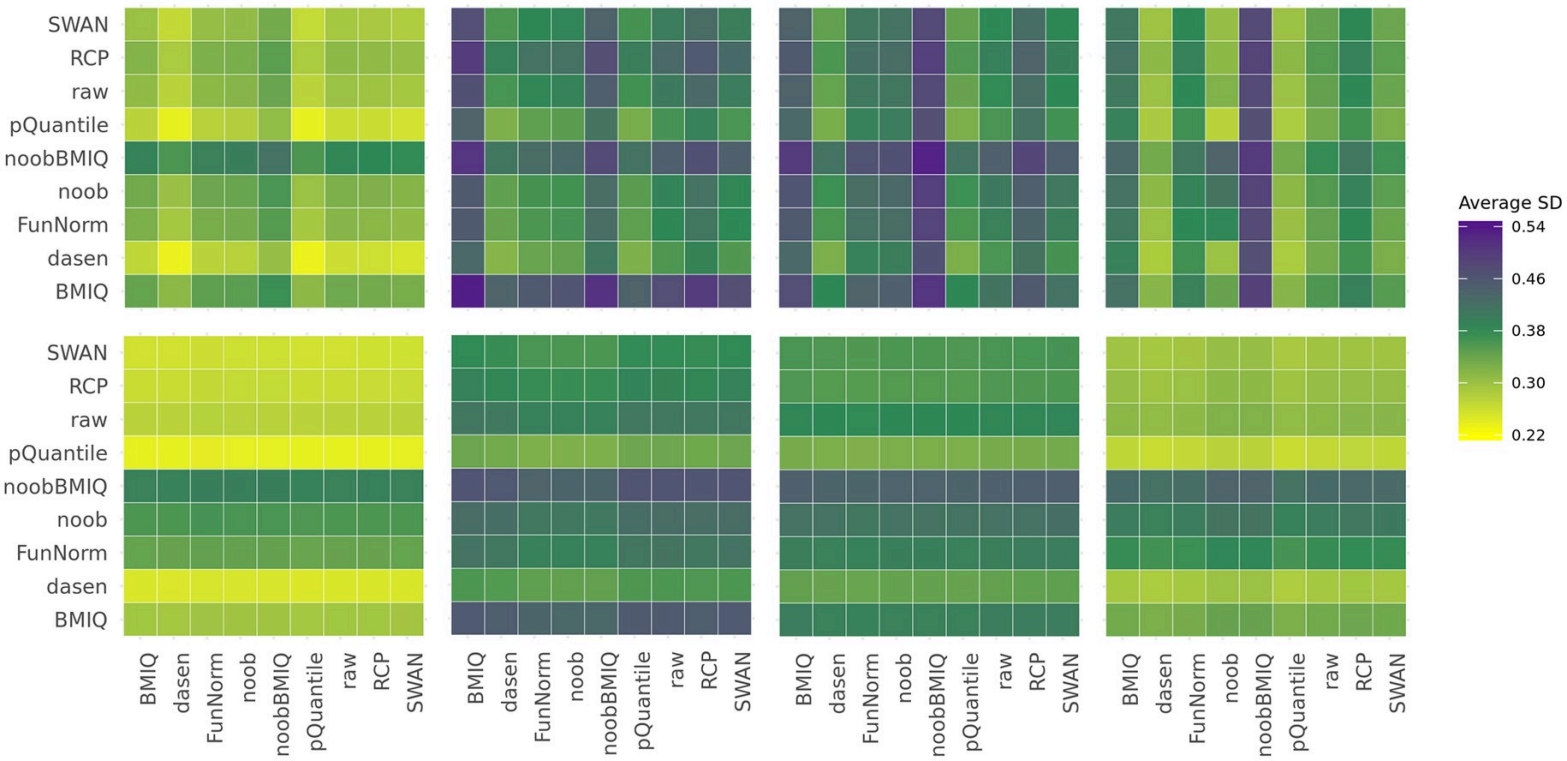
Heat maps representing the average of the M-values between-sample standard deviations. The x-axis and the y-axis indicate which pre-processing method was applied to the two datasets of the same analysis. Results are shown with (bottom) and without (top) application of *regRCPqn* and refer from left to right to datasets BloodIBD, BloodParkinson, NasalAsthma and BuccalFetalAlcohol.

The average between-sample variability depends on the pre-processing. When both datasets are pre-processed in the same way, we observe that the highest average SD is obtained when using *BMIQ* or *noobBMIQ*, while the lowest one is achieved with *dasen* and *pQuantile* (Fig 1 top, S1 Table), in agreement with the results obtained by Liu et al. [8]. Interestingly, *BMIQ* and *noobBMIQ* perform a within-sample normalization adjusting for probe type bias and, in the case of *noobBMIQ*, for the background, while *dasen* and *pQuantile* implement a between-sample normalization, a feature that could explain our results. Although absolute performances are not the aim of our study, we observe that when applying *regRCPqn*, the global between-sample variability is generally reduced, as proven by the one-tailed t-test p-values in S1 Table and shown at the bottom plot of Fig 1. This suggests that applying *regRCPqn* on top of a meta-analysis helps reducing data harmonization issues.

In particular, if the pre-processing of the two datasets is different, then the observed variance mainly depends on the method used in the first dataset, normalized using *regRCPqn* without reference distributions. This indicates that using the reference-based version of *regRCPqn* masks the effect of the pre-processing choice on the second dataset.

### Differential analysis is affected by pre-processing

We evaluated the effect of pre-processing on differential analysis by counting the number of DMPs obtained when using different methods. We considered all the nine pre-processing pipelines and we assessed the results pre-processing the two subsets of each meta-analysis both with the same method and with different ones.

Notice that the number of DMPs *per se* is not a metric of success of the method. However, since a pre-processing independent ground truth cannot be known *a priori*, the consensus among methods was used as a proxy of the robustness of the finding [27]. Therefore, we investigated the variability in the number of DMPs and the concordance between results obtained with the various methods.

**Fig 2:**
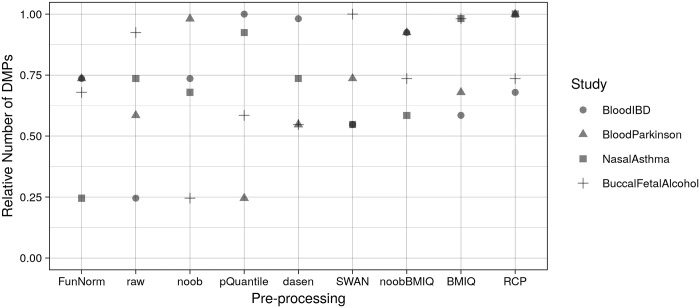
Relative number of DMPs found in each meta-analysis when using the same pre-processing for both datasets. Counts of each meta-analysis have been normalized dividing for the maximum number of DMPs. Pre-processing methods are listed on the x-axis in ascending order according to the average relative number of DMPs obtained in the four meta-analyses.

The number of DMPs found by each analysis was highly variable (from 1 to 3 orders of magnitude) depending on the pre-processing method used, as shown in Table 3. Such variability was observed even when using the same pre-processing method for both datasets (Table 4), confirming the pertinence of this analysis in the methylation meta-analysis context. Fig 2 and S2 Table show that, on average, the minimum number of DMPs was obtained when using *FunNorm*, followed by raw data. The result on *FunNorm* was in line with the findings of Liu et al. [8], showing that this method has a higher sensitivity than raw data but performs worse than other within-samples normalization methods. It was also expected for the raw data approach to identify less DMPs than other methods. In fact, the lack of a normalization step should generate more noisy data, hence the differences between cases and controls are more difficult to detect. Finally, the maximum number of DMPs was obtained with *RCP*, confirming previous findings [11].

**Table 3:** Summary statistics of the number of DMPs found by the differential analysis.

	without *regRCPqn*					with *regRCPqn*				
Study	Min	Max	Median	IQR	max-min	Min	Max	Median	IQR	max-min
BloodIBD	48	790	254	222	742	655	994	792	98	339
BloodParkinson	12	11915	1683	3861	11903	7	2569	272	405	2562
NasalAsthma	9	6208	171	1279	6199	43	1791	1196	1434	1748
BuccalFetalAlcohol	7	64	18	19	57	9	32	16	3	23

**Table 4:** Summary statistics of the number of DMPs found by the differential analysis when the two subsets of each study are pre-processed with the same method.

	without *regRCPqn*					with *regRCPqn*				
Study	Min	Max	Median	IQR	Max-Min	Min	Max	Median	IQR	Max-Min
BloodIBD	48	790	492	347	742	662	994	850	100	332
BloodParkinson	142	11915	4729	3577	11773	15	2569	465	1041	2554
NasalAsthma	27	6153	1432	2187	6126	43	1791	1636	1612	1748
BuccalFetalAlcohol	13	53	39	18	40	13	27	19	3	14

In general, our results were not stable when considering different datasets, an issue also previously reported [8]. For example, using *noob*, we observed the minimum number of DMPs in BuccalFetalAlcohol, while in BloodParkinson we obtained a number of DMPs equal to 98% of the maximum number of DMPs observed for this meta-analysis. However, sorting the pre-processing methods according to the average fraction of detected DMPs, we could observe the trend shown on the x-axis of Fig 2 (see S2 Table for numeric values).

**Fig 3:**
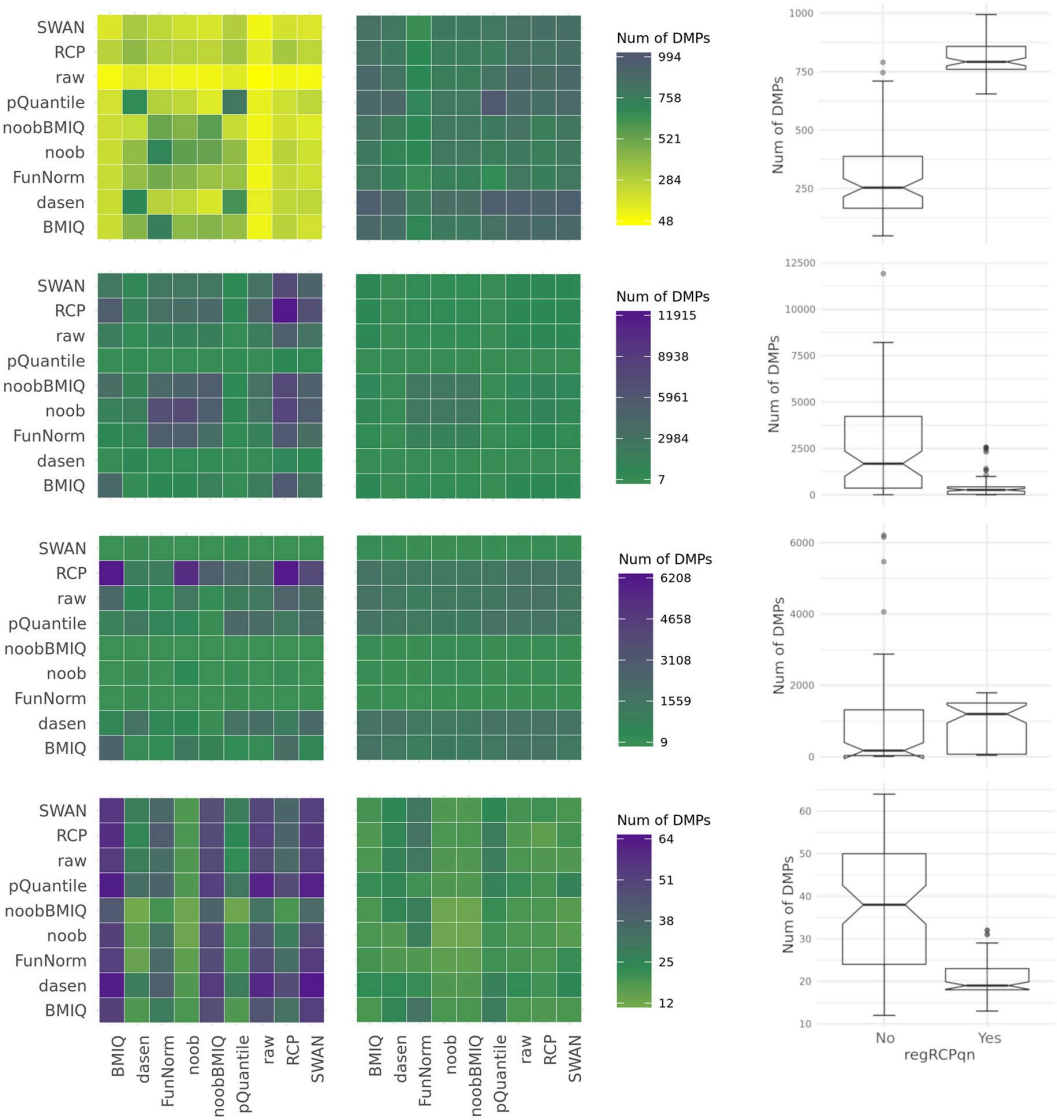
Heat maps and box plots representing the number of DMPs found in the four studies (from top to bottom: BloodIBD, BloodParkinson, NasalAsthma and BuccalFetalAlcohol) when using different pre-processing methods. For each study, the two heat maps correspond to the case where *regRCPqn* was (right) or was not (left) applied. The colour of each cell is representative of the number of DMPs found when the datasets under consideration are pre-processed according to the methods specified on the x and y axes. Here, yellow corresponds to the minimum number of DMPs (with or without *regRCPqn*), purple to the maximum and green to the median. The cells on the diagonal indicate that both datasets were pre-processed with the same method and show that DPMs remain highly variabile.

The high variability in the number of DMPs was reduced when using *regRCPqn*. In fact, Table 3 shows that the Inter Quartile Range (IQR) of the number of DMPs decreased after the application of *regRCPqn* in all meta-analyses but NasalAsthma, and that the difference between the maximum and minimum number of DMPs also decreased in all meta-analyses. The heat maps in Fig 3 graphically display these results.

In all analyses, heat maps are shown with (right) and without (left) *regRCPqn*. In general, when applying *regRCPqn*, the number of DMPs found with different pre-processing became closer to the median number of DMPs. S3 Table reports the MADs obtained with and without *regRCPqn*. Bootstrap p-values show that in all studies but NasalAsthma we obtained a lower MAD when using *regRCPqn*. Moreover, when *regRCPqn* was applied, the number of outliers was reduced in half of the datasets. These results are visually shown in Fig 3, where heat maps have lighter colours (i.e. number of DMPs closer to the median) when *regRCPqn* is used (plots on the right), and box plots have longer boxes (wider Inter Quartile Range) when *regRCPqn* is not used.

**Fig 4:**
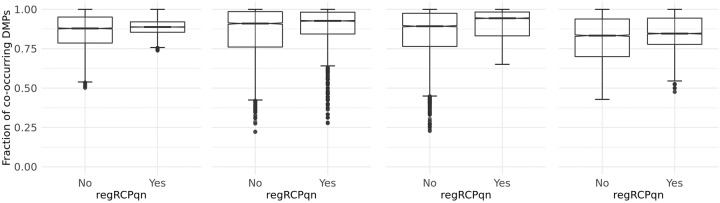
Box plots of the fraction of co-occurring DMPs computed as the number of DMPs found by one pair of analyses divided by the number of DMPs found by the analysis with less DMPs (within the pair). Results are shown for the four datasets (from left to right: BloodIBD, BloodParkinson, NasalAsthma and BuccalFetalAlcohol) and considering the cases where regRCP was or was not applied, as indicated on the x-axis.

Finally, we investigated the concordance between the results obtained with different pre-processing methods, counting the number of DMPs that are identified by more than one method. This was achieved using two approaches: first, comparing the results of each pair of pre-processing methods; second, considering the same pre-processing for both subsets of each meta-analysis and evaluating the concordance between each method and all the others. To evaluate the pairwise concordance, we considered each pair of pre-processing pipelines and computed the number of DMPs recognized by both methods (co-occurrence matrix). The box plots in Fig 4, display the distribution of the number of co-occurring DMPs considering all possible pairwise combinations of pre-processing methods with (right) and without (left) *regRCPqn*. The fraction of co-occurring DMPs varies from less than 0.25 to 1 and depends on the pair of methods and on the meta-analysis under investigation. Moreover, in general, the concordance increases using *regRCPqn*: for all datasets, one-tailed Welch’s test p-values are less than 10^−16^.

**Fig 5:**
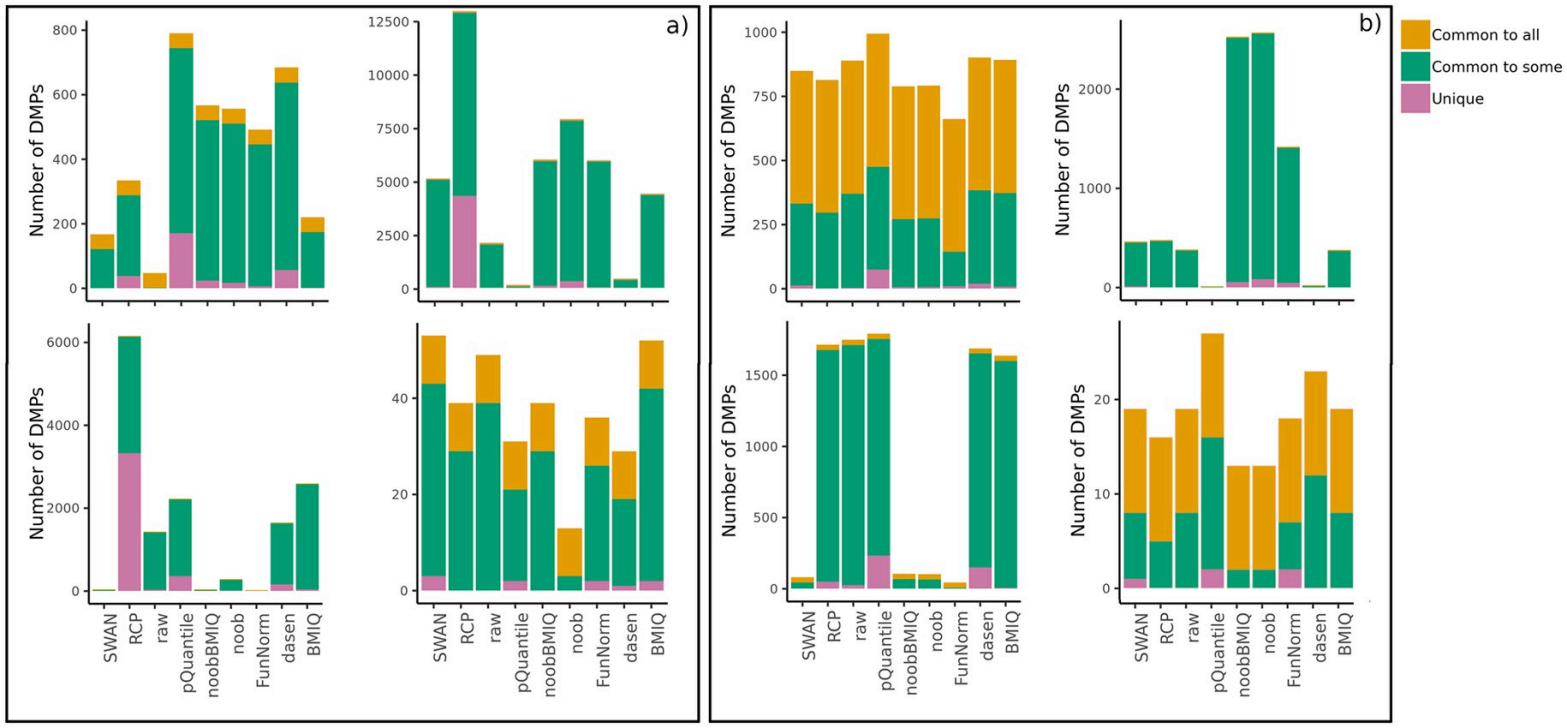
Bar plots of the number of DMPs found by each pre-processing method, considering both datasets pre-processed in the same way. Colours indicate: (i) the number of DMPs that are found by at least one other method (Common to some), (ii) by all the methods (Common to all) or (iii) only by that method (Unique). Results are shown for the four analyses: BloodIBD (top-left), BloodParkinson (top-right), NasalAsthma (bottom-left), BuccalFetalAlcohol (bottom-right). Panel a) refers to the case where *regRCPqn* is not applied, while panel b) shows the results obtained using *regRCPqn*.

The concordance between each method and all the others was evaluated by computing the number of DMPs that are found by all, some or none of the other approaches. S4 Table and the bar plots in Fig 5 show for each method the number of identified DMPs that are also detected by all, some, or none of the other methods. Without using *regRCPqn* (Fig 5, panel a)), all meta-analyses contained a subset of DMPs that were identified by all methods and *unique* DMPs were obtained mainly by methods that identified a greater number of DMPs (*RCP* and *pQuantile*). One-tailed Welch’s test p-values (S4 Table) suggested that applying *regRCPqn* improves the outcome. In fact, even if the statistical significance was low for two out of four datasets, our results showed that the use of *regRCPqn* tends to increase the number of DMPs shared by all methods while decreasing the number of *unique* DMPs identified by only one method (Fig 5, panel b).

Our results, hence, suggested that harmonizing the data with *regRCPqn* reduces the variability related to the use of different pre-processing procedures in the two datasets.

## Conclusion

The high availability of public DNA methylation datasets opens to the possibility of performing meta-analyses. Often, however, data are pre-processed in different ways, due to the lack of consensus on pre-processing and on potentially different needs associated to the specificities of the study. Further, owing to the limited compliance to GEO guidelines and standards, raw data are seldom made available. In order to assess the impact of this on further analyses we systematically investigated the effects of different pre-processing methods. We found that pre-processing affects both the between-sample variability and the results of meta-analysis, using as exemplar differential analysis. In general, results are highly variable depending on the dataset under investigation. We must notice that our results are based on a selection of dataset sufficiently diverse to grant generalization, but limited (4 dataset pairs) and that dataset agreement is used as a performance metric, as ‘ground truth’ is not available for this type of analyses. Nevertheless, we were able to identify some general trends. Concerning the between-sample variability, we show that the lowest variability among control samples is achieved when pre-processing methods that perform a between-sample normalization are applied (i.e. *dasen* or *pQuantile*), while the highest variability is obtained when using *BMIQ* or *noobBMIQ*. The dependence on the pre-processing method was also observed for the results of the differential analysis. Specifically, the number of DMPs identified in the comparison between cases and controls can vary by more than a few thousands when changing the pre-processing method, and the concordance between the identified DMPs is only partial.

Data harmonization remains a main issue of meta-analysis. Our algorithm, named *regRCPqn*, largely relies on *RCP* and can reduce the global variability between control samples and increase the stability of the differential analysis.

## Supporting information

S1 Table For each combination of pre-processing, we show the between-sample variability (average SD) with and without *regRCPqn*. T-test was performed to test if the difference in average SD using *regRCPqn* or not is statistically significant.
(XLSX)

S2 Table For each pre-processing we report the number of DMPs found in each meta-analysis. Mean and standard deviation of the number of DMPs across the 4 studies are also reported. Only the case in which the same pre-processing is used for both datasets of the meta-analysis is considered.
(XLSX)

S3 Table For each dataset, we report the MAD of the number of DMPs obtained when using different preprocessing. Outliers and boostrap p-values that tests whether using *regRCPqn* reduces MAP, are also reported.
(XLSX)

S4 Table For each dataset, we report for each pre-processing method the number of DMPs also found by all the others (Common to all), by at least one other (Common to other) and by none of the others (Unique). To test whether using *regRCPqn* increases the number of DMPs common to all methods and decreases the number of *unique* DMPs, we considered the difference between the fractions obtained with and without *regRCPqn* and we used a one-tailed Welch’s test. Finally, we report a summary table in which the number of DMPs obtained for each dataset and for each pre-processing method, using and not using *regRCPqn*, is indicated. Only the case in which the same pre-processing is used for both datasets of the meta-analysis is considered.
(XLSX)
